# Automated Neuron Tracing Using Content-Aware Adaptive Voxel Scooping on CNN Predicted Probability Map

**DOI:** 10.3389/fnana.2021.712842

**Published:** 2021-08-23

**Authors:** Qing Huang, Tingting Cao, Yijun Chen, Anan Li, Shaoqun Zeng, Tingwei Quan

**Affiliations:** ^1^Britton Chance Center for Biomedical Photonics, Wuhan National Laboratory for Optoelectronics-Huazhong University of Science and Technology, Wuhan, China; ^2^MoE Key Laboratory for Biomedical Photonics, Collaborative Innovation Center for Biomedical Engineering, School of Engineering Sciences, Huazhong University of Science and Technology, Wuhan, China

**Keywords:** neuronal image, tubular object tracing, content-aware adaptive voxel tracing, 3D CNN, high precision

## Abstract

Neuron tracing, as the essential step for neural circuit building and brain information flow analyzing, plays an important role in the understanding of brain organization and function. Though lots of methods have been proposed, automatic and accurate neuron tracing from optical images remains challenging. Current methods often had trouble in tracing the complex tree-like distorted structures and broken parts of neurite from a noisy background. To address these issues, we propose a method for accurate neuron tracing using content-aware adaptive voxel scooping on a convolutional neural network (CNN) predicted probability map. First, a 3D residual CNN was applied as preprocessing to predict the object probability and suppress high noise. Then, instead of tracing on the binary image produced by maximum classification, an adaptive voxel scooping method was presented for successive neurite tracing on the probability map, based on the internal content properties (distance, connectivity, and probability continuity along direction) of the neurite. Last, the neuron tree graph was built using the length first criterion. The proposed method was evaluated on the public BigNeuron datasets and fluorescence micro-optical sectioning tomography (fMOST) datasets and outperformed current state-of-art methods on images with neurites that had broken parts and complex structures. The high accuracy tracing proved the potential of the proposed method for neuron tracing on large-scale.

## Introduction

Digital reconstruction or tracing of neurons, which converts a neuronal image into a digital representation by obtaining the 3D spatial position of neuron skeletons and building their topological connections, is one of the major subjects in computational neuroscience (Parekh and Ascoli, 2013). Neuron tracing is regarded as the basis of the neuronal morphological study, neuron phenotype identification, and neural circuit building, which is important in understanding the brain organization and function in diseases like Alzheimer and Schizophrenia (Economo et al., 2016; Lin et al., 2018). However, current neuron tracing is mainly performed by hand, it could take hours of hard work to trace a simple dendritic tree and months of labor for large-scale neurons (Economo et al., 2016). The lack of automatic and accurate tracing method has become a critical technical bottleneck in neuroscience research.

**Figure 1:**
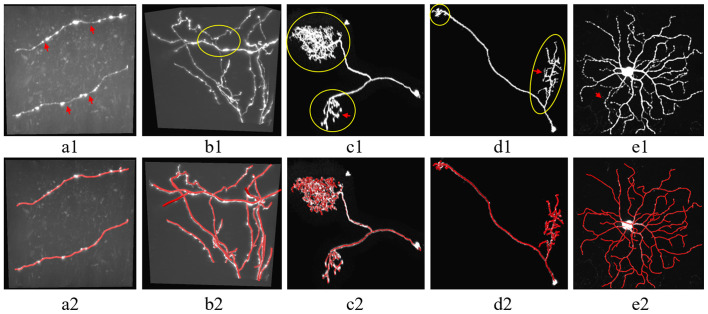
Examples of 3D optical neuronal images with complex tree-like distorted structures and broken parts of the neurite. **(A1,B1)** Images from fluorescence micro-optical sectioning tomography (fMOST) datasets. **(C1–E1)** Images from BigNeuron datasets. **(A2–E2)** Corresponding manual tracing results of **(A1–E1)**. Yellow circles point out some twisted structures with various directions. Red arrows point to some broken neurite parts with the sudden change of intensity.

Neuron tracing from optical microscopy images was challenging. First, the high artifacts and noises in the neuronal image fuzzy the neurites (see Figure 1), and easily lead to inaccurate tracing. Second, the neuron structure is complex and distorted with various direction changes, even an experienced annotator had to spend hours to trace these tree-like or mushroom-like structures from the image (Figures 1B1–E1). Third, the intensity distribution of neurites is often inhomogeneous and plenty of broken neurite parts exist due to the sudden intensity changes along the neurites, which leads to an interruption of neuron tracing (as pointed out by arrows in Figure 1). These are also common problems of tubular objects tracing of medical images, including retinal, liver vessel, and brain vessel tracking.

Currently, numerous semi-automatic or automatic algorithms have been proposed for neuron tracing. APP2 used the image intensity and space information for gray-weighted image distance-tree calculation and applied hierarchical pruning for refinement (Xiao and Peng, 2013). Open-Snake traced the neurites by adapting open-curve snake based on the combination of a gradient vector flow, neurite orientations, and a set of control rules (Wang et al., 2011). neuTube applied a model-based algorithm for neuron tracing using the local geometrical and global structures of neurite (Zhao et al., 2011; Feng et al., 2015). Tubularity-Flow-Field realized neuron tracing *via* centerline extraction on segmentation achieved by applying directional regional growing based on the direction of neurite tubularity (Mukherjee et al., 2015). RPCT, NeuroGPS, and SparseTracer used principal curves tracing, and some used direction constrains for reconstruction (Bas and Erdogmus, 2011; Quan et al., 2016; Li et al., 2017b). FMST adopted fast marching and minimum spanning tree for faster tracing without loss of small branches (Yang et al., 2019). Some smart machine-learning-based methods have also been applied for neuron tracing. SmartTracing (Chen et al., 2015) and ST-LVF (Li et al., 2019b) first extracted different local features based on the tracing results of existed algorithms (APP2 and SparseTracer respectively), then applied support vector machine (SVM) to promote complete neuron tracing from noisy image. These algorithms combined the advantages of various image processing methods by considering different neurite characters for neuron tracing. They were usually sensitive to parameters setting and hard to trace these complex-structure and broken neurons. Besides, the self-learning smart algorithms relied on previous tracing and were computationally complicated and time-consuming (Chen et al., 2015; Li et al., 2019b).

Deep convolutional neural network (CNN) automatically extracted more discriminative image features and outperformed traditional algorithms in image segmentation (Çiçek et al., 2016; Li et al., 2017a; Chen et al., 2018). The technique was also employed in neuron tracing. DeepNeuron applied 2D CNN for neurite detection and connection, and showed low performance on neurite tracing from 3D noisy image (Zhou et al., 2018). Li et al. (2017a) applied 3D CNN to suppress the high noise to improve the tracing performance of the previous tracing method of APP2. Recently, weakly-supervised learning was developed to allow automatic training labels’ generation to promote deep learning usage in neuron tracing (Huang et al., 2020). From these previous works, we found they still had difficulties in tracing these neurites with distorted and branches structures or broken parts. These methods generally use the strong prediction of CNN, such as the prediction by maximum classification, and some limited setting rules for neuron tracing and graph tree building. Thus, their performance decreased when tracing these above hard neurites.

In the article, we proposed an automatic and accurate method for neuron tracing to address the difficulties of neurons with complex structures and uneven intensity distribution. We estimated the neurite probability map from a noisy image by 3D residual CNN. Unlike previous methods, we took full utilization of the probability characters and proposed a content-aware adaptive voxel scooping method for continuous tracing of broken neurites, which is based on the distance, connectivity, and directional probability continuity. We evaluated the proposed method on the public BigNeuron datasets (Peng et al., 2015) from different organizations and fluorescence micro-optical sectioning tomography (fMOST) datasets (Gong et al., 2013). Our method achieved comparable results of manual tracing and was superior to some current algorithms on neuronal images with complicatedstructure and broken neurites.

## Materials and Methods

**Figure 2:**
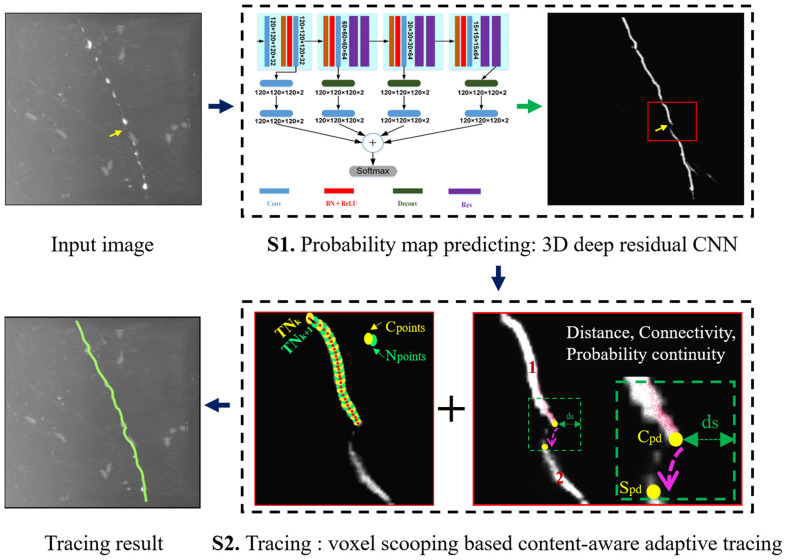
Flowchart of the proposed neuron tracing method.

The flowchart of the proposed neuron tracing method is shown in Figure 2. It includes two steps: (1) Preprocessing: predicting neurite probability map by 3D deep residual CNN. (2) Continuous neurite tracing of broken parts on the probability map by voxel-scooping (VS) based content-aware adaptive tracing (CAAT).

### Preprocessing: Probability Prediction by 3D Residual CNN

In preprocessing, the neurite probability is estimated by 3D CNN, considering its ability in automatic feature extraction, to detect the fuzzy neurite from a noisy image. Instead of using the very complicated network module (more than 200 layers) in Li et al. (2017a), the 3D deep voxelwise residual network (VoxResNet, 25 layers; Chen et al., 2018) is employed as our segmentation model for its simple and elegant structure, deep supervised mechanism, and outstanding predictive ability. The detailed network architecture is referred to in Chen et al. (2018).

In the training, randomly cropped image samples with overlapped regions and size 120 × 120 × 120 or 64 × 64 × 64 are used for training considering the computational power and accuracy. To avoid serious imbalanced classes between the foreground (neurite) and background, samples with few foreground voxels are abandoned. To increase the diversity of samples and improve network robustness, we apply random rotation, flip, contrast, brightness adjustment, and Gaussian blur for data augmentation.

To further prevent the prediction bias of imbalanced classes and improve accuracy, we combined the dice loss and weighted cross-entropy loss as the hybrid loss (Huang et al., 2020), which is defined as follows:

(1) $$loss_{\text{hybrid}}=(1-2\frac{\sum_{i=1}^{m}p_{i}g_{i}+\varepsilon }{\sum_{i=1}^{m}p_{i}+\sum_{i=1}^{m}g_{i}+1})+\sigma (\sum_{i=1}^{m}-\frac{\sum_{i=1}^{m}g_{i}}{m}\cdot g_{i}log(p_{i}))$$

Where *P*_i_ is the predicted probability of pixel *i*. *g*_i_ is the label value with 1 for foreground and 0 for background. σ is the weighted parameter and set to 0.5 in the experiment.

### Neuron Tracing

With proper training, CNN could achieve expert-level performance on neurite prediction and even had low predictions for the fuzzy and broken neurites in a noisy background. Here, we take full usage of the CNN predicted probability using object’s internal properties and probability continuity, and propose an automatic neuron tracing method. The method includes four steps: (1) Initial segmentation of probability map; (2) Seed point selection; (3) Neurite tracing using VS based CAAT; and (4) Neuron tree graph establishing and branch pruning for simplicity.

The initial neurite segmentation should remain good neurite shape meanwhile contain more neurite parts. An initial threshold estimation method based on the intensity distribution and heuristic rules of the probability is applied for better performance than maximum classification.

First, we estimate the adaptive threshold of the neurite probability predicted by CNN, instead of using the typical threshold 0.5 to classify the foreground from the background of the prediction. A Gaussian function is used to fit the background probability histogram of the prediction, the histogram with probability <0.5, to estimate the mean *μ*_bg_ and standard deviation *σ*_bg_ of the background probability. Then, the adaptive threshold is set to *μ*_bg_+λ*σ*_bg_. Where *λ* is set to 3 to exclude more background noise meanwhile keep most foreground signals.

The first points of every connected binary region are selected as seed points for the subsequent automatic tracing. In tracing, the skeleton points (or tracing nodes) of the neurites and their connection relationships are built. We trace the neurites from the predicted probability using VS based CAAT. The VS tracing approach starts from the seed points inside the object, and iteratively generates new voxel clusters around the previous cluster along the object based on the region connectively and spatial position. The searched clusters are then used to produce the skeleton node of the object and build the spatial connection of the nodes. For precise and fast tracing, we apply VS for normal tracing of the connected neurite regions on initial segmentation *I*_seg_, and CAAT for continuous tracing of broken neurite fragments based on the probability image *P*_img_.

Here, we define the current point set as *C*_ps_, and the next point set as *N*_ps_. The center point of *C*_ps_ and *N*_ps_ are defined as the current tracing node *TN*_k_ and the next tracing node *TN*_k+1_, respectively. For normal tracing, *N*_ps_ are searched by VS using *C*_ps_ as seed point set based on 26-connected domain and scooping distance. The maximum distance of *TN*_k_ and unvisited object voxels in the 26-connected neighborhood of *C*_ps_ is calculated as the scooping distance. Any of the unvisited object voxel that falls into the ball of the scooping distance of *TN*_k_ is searched and added into *N*_ps_ for accurate estimation in case of directional changes. For discontinuous tracing, if *N*_ps_ couldn’t be searched by VS, the new point sets *S*_ps_ is then searched by CAAT and used as *N*_ps_ [detailed process of CAAT is in Section “Content-Aware Adaptive Tracing (CAAT)”]. The searched *N*_ps_ is used to update *C*_ps_ iteratively, and the tracing continues until no new points are searched.

After tracing, the tree graph of the neurites is built based on the neighborhood connection relationship and spatial position of the traced skeleton nodes. Three kinds of nodes are defined to build the tree graph: leaf node, path node, and branch node. Leaf node exists at the end of a neurite that with only one immediate neighboring node. Path node exists between the leaf node and branch node along the neurite path with only two immediate neighbors. Branch node is the intersect point of crossed branches with more than two immediate neighbors (Li et al., 2019a). In the graph tree building, we apply the length first criterion. The nearly longest neurite path in each individual tree is first chosen out as the stem, which starts from the seed point and ends at the last node with nearby nodes linked by line based on their connection relationship. Other neurite paths that form the branch nodes of the truck to the leaf nodes are defined as the tree branch. To remove some spurious end nodes that are caused by neuronal irregularities such as the expansion of neurite part or bouton, we prune short branches. If the skeleton point number from a leaf node to a branch node of a neurite path is less than *l*_num_, the branch is pruned. *l*_num_ can be set between 5 and 10, and is set to 6 in the experiment. Finally, the established neuron tree graph is saved in SWC file format that records the nodes’ sequence, position, and connection, and can be accessed by the commercial software Amira and Neurolucida.

### Content-Aware Adaptive Tracing (CAAT)

For expert observers, the distance, region connection attributes, and probability properties between neurite fragments are combined to determine their connection relationship. In the article, we simulate the judging process of the experts and propose the CAAT method by using three content perception terms (distance, connectivity, and direction probability continuity term) for connection. Detailed processing steps are described in Algorithm 1.

#### Distance Term

The distance between nearby broken parts of an object should be in a certain range. A closer distance between current points *C*_ps_ and searched points *S*_ps_, a higher connection probability. The term is given by:

(2) $$d_{\text{score}}=\{\begin{array}{cc} 1 & ;\;d\leq d_{\text{t}}\\ \exp(-\frac{d-d_{t}}{3}) & ;\;d>d_{\text{t}} \end{array}$$

Here, we find the closest point pairs (*c*_p_, *s*_p_) of *C*_ps_ and *S*_ps_ by traversal search. $d={\left\|c_{\text{p}}-s_{\text{p}}\right\|}_{\infty }$ is the Chebyshev distance between the point pairs. *d*_t_ is the distance parameter and also the only parameter of CAAT. It is set to 4–5 pixels based on experience to prevent over-connection of nearby neurites or under-connection of discontinuous neurites.

#### Connectivity Term

To prevent repeated tracing caused by the trace of unvisited points that belong to the same connected domain of *C*_ps_ (like neurite irregularities), *C*_ps_ and *S*_ps_ must belong to different connected domains (region 1 and 2 in Figure 2 S2), and *C*_ps_ ⊆ *R*_i_, *S*_ps_ ⊆ *R*_j_. The term is defined:

(3) $$c_{\text{score}}=\{\begin{array}{cc} 0; & R_{i}=R_{j}\\ 1; & R_{i}\neq R_{j} \end{array}$$

Where *R*_i_ and *R*_j_ are the 2- connected domains that contain *C*_ps_ and *S*_ps_, respectively.

#### Direction Probability Continuity Term

Various image content and appearance features are combined into the CNN estimated neurite probability. For broken ones, the probabilities along the direction of $\vec{c_{\text{p}}s_{\text{p}}}$ are higher, the connection probability is larger. The term is calculated:

(4) $$dpc_{\text{score}}=\exp(-(d_{\text{ps}}-\sum CP(M_{\text{ps}})/d_{\text{ps}}))$$

Where *CP* is a continuity probability function that modifies the neurite probability. *M*_ps_ are connected points along the line of $\vec{c_{\text{p}}s_{\text{p}}}$. *d*_ps_ is the voxel number of *M*_ps_.

(5) $$CP(i)=\{\begin{array}{cc} 1, & P_{\text{img}}(i)>t_{1}\\ P_{\text{img}}(i), & P_{\text{img}}(i)\leq t_{1} \end{array}$$

Here, *t*_l_ is a low threshold used to discover more possible neurite voxels between *c*_p_ and *s*_p_, and excludes at least 50% background noise. It is estimated based on the background probability histogram.

(6) $$\min\left(\sum_{k}{\left\|P_{\text{img}}(k)\leq t_{1}\right\|}_{0}\geq 0.5\sum_{k}{\left\|P_{\text{img}}(k)\leq 0.5\right\|}_{0}\right)$$

And refined as *t*_l_ =min(0.1, *t*_l_). Finally, the adaptive linking score is built based on the three above content terms.

(7) $$link_{\text{score}}=d_{\text{score}}\cdot c_{\text{score}}\cdot dpc_{\text{score}}$$

When *link*_score_ > 0.5, *S*_ps_ is used as *N*_ps_ for continuous tracing of broken neurites.

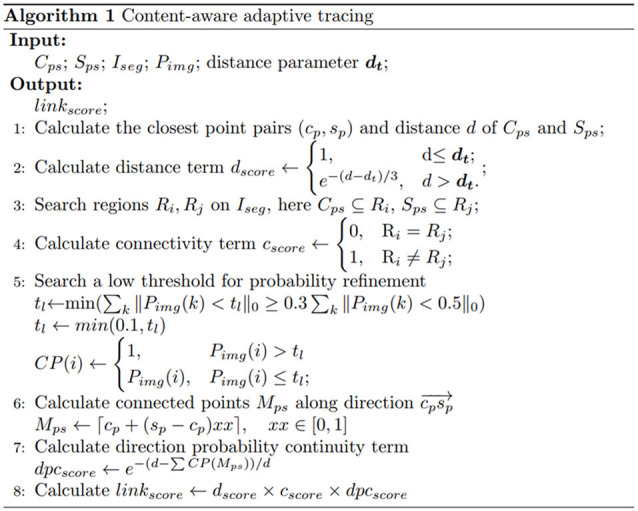



### Evaluation Metrics

The typical metrics of precision and recall are used for objective evaluation of the proposed and current novel neuron tracing methods (Quan et al., 2016; Li et al., 2017b). We first equally resampled the skeleton points to keep the same distance of adjacent points (1 pixel). Here, the manual tracing results are used as the gold standard. If the closest distance of a traced skeleton point by the algorithm to the gold standard is less than 6 pixels, the point is defined as a true positive (TP) point (Li et al., 2017b). The parameter was set to 6 in our application, as we resampled the skeleton points to the same size and the experiment was mainly performed on neuronal images that contained axon neurites or dendrite and axon with similar radius (generally 4–8 pixels). The precision and recall rates kept stable when the parameter ranged from 6 to 10 pixels (1 μm/pixel; Quan et al., 2016), and 6 was reasonable in our experiment. Precision and recall are used to reflect tracing accuracy and integrity and calculated as the number of TP points to the number of skeleton points obtained from algorithms and manual tracing, respectively. FN means false negative skeleton points.

(8) $$\begin{array}{ccc} precision & = & TP/(TP+FP)\\ recall & = & TP/(TP+FN) \end{array}$$

The morphological parameters including the neuron path lengths and branch points are also calculated in the study for evaluation of neuron morphology.

### Experimental Setup

The public BigNeuron datasets (Peng et al., 2015) and private fMOST datasets (Gong et al., 2013) were used for evaluation. This study focuses on precise tracing on discontinuous neurites (mainly axons, as the dendrites were generally easy to identify with brighter and thicker neurites than axons). Therefore, we selected 38 neuronal images from BigNeuron dataset that had the same bit depth (0–255), available standard reconstruction, and contained noises, rich axons with thin, dim, and broken neurites as our training (30 subjects) and testing datasets (eight subjects). These neuron images were from different organizations or projects including the Utokyo, Janeliar Fly Light, Allen Institute, and Taiwan FlyCircuits. They contained various species including cells of fly, mice, and humans. They were generated using different imaging methods including confocal and 2-photon. Their image resolutions were between 0.1–0.593 μm × 0.1–0.593 μm × 0.3–2 μm, and image sizes were between 511–2,048 × 511–2,048 × 11–537. The fMOST image was generated using micro-optical sectioning tomography imaging (Li et al., 2010) on a C57BL/6J mouse brain labeled by AAV virus in the cortex. The image resolution was 0.2 μm × 0.2 μm × 1 μm. We selected 48 image blocks that contained abundant and discontinuous axon neurites from the fMOST dataset as training set. Their image sizes were 300 × 300 × 300. We tested the algorithm on 16 image blocks with sizes between 164–1,000 × 214–1,000 × 300. These training and testing image blocks were captured from different brain areas. We also evaluated the proposed method on a large-scale neuronal image with image size of 3,630 × 3,630 × 1,080. In the experiment, the 3D CNN was implemented on Pytorch, and the tracing method was implemented on Matlab. The CPU was Intel i7-6850K (64 GB RAM) and GPU was NVIDIA 1080Ti.

## Results

**Figure 3:**
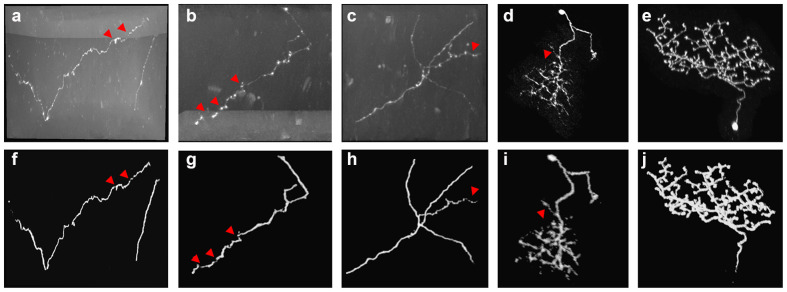
Examples of 3D CNN prediction **(F–J)** on noisy optical images **(A–E)** with complex-structures and broken neurites. Red arrows point to the broken neurite parts on the original image and corresponding probability.

In the proposed method, neurite probability is estimated by 3D residual CNN as preprocessing. Figure 3 shows the prediction performance on neuronal images that have high noises, complicated twisted structures and broken neurite fragments. As we can see, the trained CNN effectively reduced the high artifacts and noises of the optical images. These neurons with unevenly distributed intensities along the neurites and various twisted structures and branches were generally predicted by the CNN accurately (Figures 3F–J). Only for these hard parts that had broken fragments or inhomogeneous areas that with sudden intensity and direction changes, the predictions seemed to be discontinuous and there still had some low predictions for these parts, which was quite similar to the estimated probability of humans for uncertain objects. The comparison in Figures 5–8 further illustrated the network effectiveness on these images. The results proved that the presented network was precise and robust in neurite prediction from noisy images.

**Figure 4:**
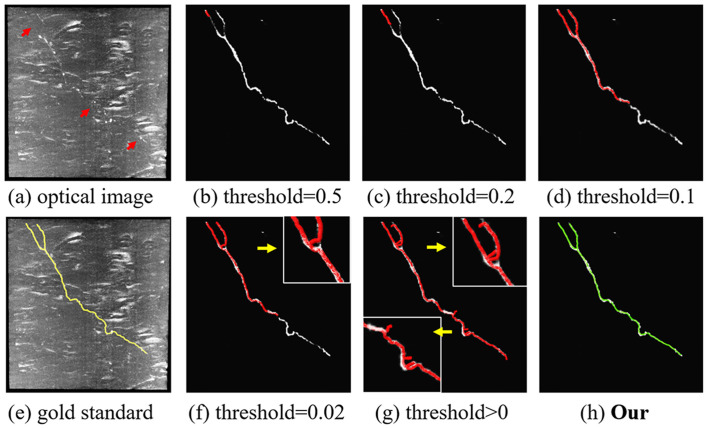
Comparison of the proposed content-aware adaptive tracing (CAAT) **(H)** and typical threshold-based classification method **(B–D,F,G)** on neuron tracing with the same initial seed point. Optical image **(A)**; gold standard **(E)**. Inaccurate traced areas were amplified in the white box.

**Figure 5:**
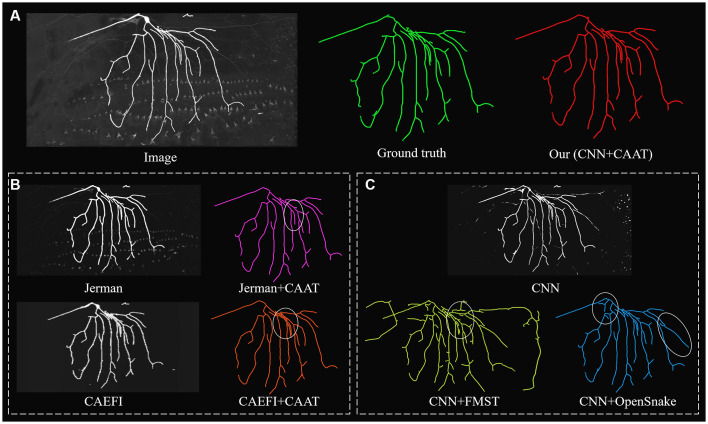
Comparative performances of ablation studies on a complex and noisy big neuron image to illustrate the role of CNN prediction and CAAT of the proposed method, by applying current novel enhancement methods [Jerman filter (Jerman et al., 2015) and CAEFI (Jeelani et al., 2019)] to replace CNN prediction **(B)** and tracing methods [FMST (Yang et al., 2019) and OpenSnake (Wang et al., 2011)] to replace CAAT **(C)** alternatively. Comparative tracing results of gold standard and our method **(A)**. Inaccurate traced areas were marked by white ellipses.

**Figure 6:**
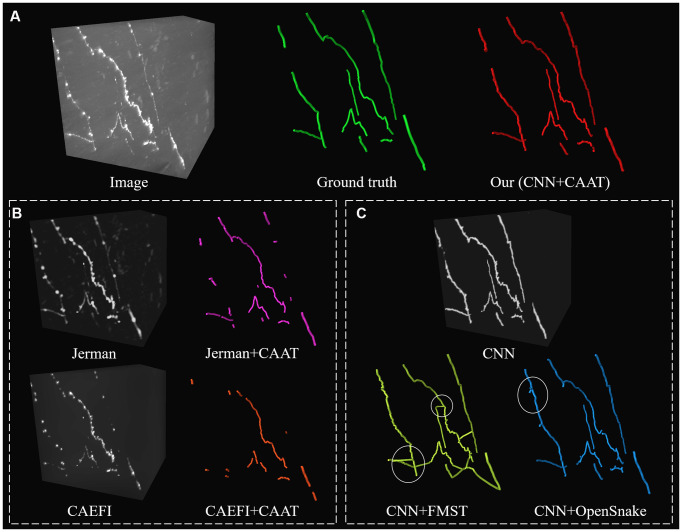
Comparative performances of ablation studies on a discontinuous and noisy fMOST image to illustrate the role of CNN prediction and CAAT of the proposed method, by applying current novel enhancement methods [Jerman filter (Jerman et al., 2015) and CAEFI (Jeelani et al., 2019)] to replace CNN prediction **(B)** and tracing methods [FMST (Yang et al., 2019) and OpenSnake (Wang et al., 2011)] to replace CAAT **(C)** alternatively. Comparative tracing results of gold standard and our method **(A)**. Inaccurate traced areas were marked by white ellipses.

**Figure 7:**
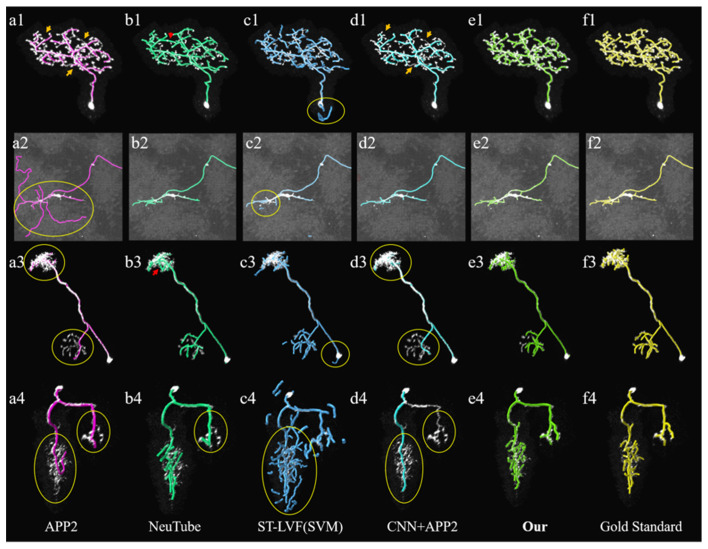
Comparative performances of the proposed method and current novel algorithms on the public BigNeuron datasets. **(A1–F4)** Corresponding tracing results of different novel methods, our method and gold standard. Inaccurate traced areas were highlighted with yellow.

**Figure 8:**
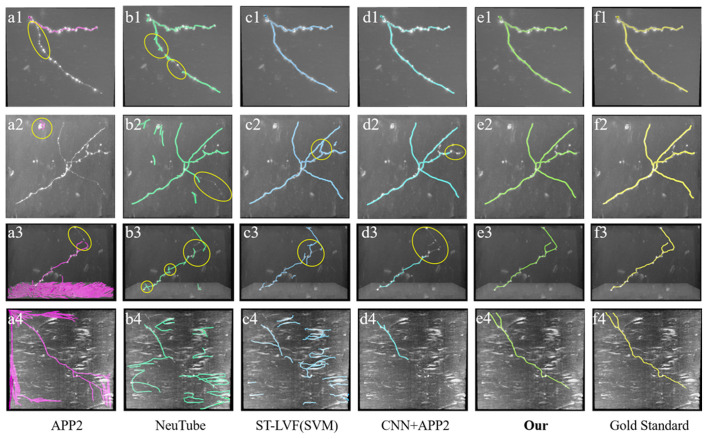
Comparative performances of the proposed method and current novel algorithms on fMOST datasets. **(A1–F4)** Corresponding tracing results of different novel methods, our method and gold standard. Inaccurate traced areas were highlighted with yellow.

To illustrate the effect of the proposed CAAT in neuron tracing of broken parts and complex structures, we compared the performance of CAAT and VS using the typical threshold-based probability classification method (see Figure 4). The probabilities for broken neurite parts were discontinuous. A higher classification threshold would lead to a severe fracture phenomenon. A lower classification threshold would make the tracing result be interfered with by the background noise and deviated from normal tracing at complex neurite structures, such as bifurcation, distortion, and turning. A proper threshold for probability classification was hard to be found due to the abundant appearances of an image with uneven intensities. Using adaptive tracing strategy, the performance of CAAT was nearly the same as the manual tracings and outperformed VS tracing using threshold-based classification method on predicted probability map.

We further performed an ablation study to illustrate the effectiveness of CNN prediction and CAAT of the proposed method. For validation of CNN prediction effectiveness, current typical and novel enhancement methods [Jerman (Jerman et al., 2015) and CAEFI (Jeelani et al., 2019)] were applied to replace CNN prediction for the next tracing by CAAT. Jerman filter was an improved multiscale vesselness filter for objects with different radii and could be used for neurite enhancement considering their tubular structures (Wang et al., 2011; Basu et al., 2013; Zhou et al., 2015). CAEFI (Jeelani et al., 2019) was a novel enhancement method for image containing filamentous structures, by combining the characteristics of gradient sparsity and filamentous structure constraint for effective removal of high noises. The comparisons were performed on both the BigNeuron and fMOST images that contained high noises, complex neuron structures, and discontinuous neurites with different radius (see Figures 5, 6B). For good enhancement performances, the parameters of Jerman and CAEFI were manually adjusted. The scale parameters of Jerman were set to 1–3 or 1–4, and the cutoff threshold was set to 0.75 or 0.6, respectively. The window size of CAEFI was set to 9, and the smooth parameter was set to 0.002 and 0.0001 for BigNeuron and fMOST images, respectively. As seen in Figures 5, 6B,C, the Jerman, CAEFI and CNN prediction all suppressed high noises of neuronal images. Jerman filter enhanced neurites with different radii. The filter would thicken the neurites and overlapped nearby neurites, and led to the missing tracing of close neurites (Figure 5B). Jerman filter also showed low performances on discontinuous neurites for the weak estimation at uneven objects, and large fragments existed (Figure 6B). CAEFI effectively removed cluster and noise and obtained a clear background. The boundary of the neuron showed hairy structures and led to over-tracing of close neurites (Figure 5B). The gradient smooth constraint strategy wiped up the thin or unobvious objects due to their indistinctive gradients and caused the discontinuous tracing of uneven neurites (Figure 6B). CNN showed a clear prediction of complex structures and discontinuous neurites, promoted proper neurite tracing by CAAT method, and outperformed the Jerman and CAEFI enhancement methods in these cases.

For validation of CAAT effectiveness, automatic and state-of-art tracing methods [FMST (Yang et al., 2019) and OpenSnake (Wang et al., 2011)] were applied to replace CAAT after CNN prediction. To deal with discontinuous neurites, FMST first adapted the over-reconstruction strategy of neurons, and merged broken neurites after the pruning step (Yang et al., 2019), OpenSnake realized automatic snake merging after branching point detection (Wang et al., 2011). These methods proved good performance on discontinuous neurite tracing, while over-traced or over-connected to some nearby noises or neurites in some cases (Figures 5, 6B). CAAT made a more proper judgment than the two tracing methods to decide whether two broken fragments should connect or not, and achieved fine tracing of the neuron with no connection to nearby neurites. As marked out in Figure 5, CAAT could more accurately trace close neurites than FMST, and get the correct connection relationship of nearby or branching neurites. These performances proved that the CNN prediction and CAAT both achieved similar or better performances than current novel enhancement or tracing methods, respectively. The proposed method combined the advantages of CNN prediction and CAAT, and obtained identical tracings of manual tracing results, especially for neurons with complex structures or uneven intensity distribution along neurites.

We compared the performances of the proposed method and current novel algorithms on the public BigNeuron datasets, including the typical APP2 (Xiao and Peng, 2013), neuTube (Feng et al., 2015), machine-learning based ST-LVF (Li et al., 2019b), and CNN+APP2 algorithm similar to Li et al. (2017a), which was realized by applying APP2 on the adjusted neuronal image using CNN predictions. As shown in Figure 7, the neuronal morphologies were complicated in BigNeuron datasets, which had mushroom-like structures, lots of twisted neurites, and uneven intensity distributions. On high noisy images, APP2 and ST-LVF algorithms, which achieved the initial segmentation by using threshold-based methods, misidentified the background noise as the signals and lead to large tracing errors of the noises. On images with complex neuronal structures, APP2 was hard to reconstruct the precise morphologies, it missed a lot of neurite branches and went in the wrong direction of the twisted structures. neuTube missed some neurite branches and wrongly connected some separated nearby neurites. ST-LVF tended to over-reconstruct the twisted structures and generated lots of wrong branches. CNN+APP2 also missed lots of twisted neurites as a result of APP2 algorithm. On images with uneven neurites and broken parts, APP2, neuTube, and CNN+APP2 algorithms failed in the tracing of some broken parts which caused large incomplete neurite tracings. The proposed method can trace the precise neuron morphology even in noisy images with discontinuous neurites and complex neuronal structures. As proved in Table 1, the average precision of our method was 98.2%, which was similar to the results of CNN+APP2, and better than that of APP2, neuTube, and ST-LVF. The average recall of our method was 95.1%, which was vastly better than that of APP2, neuTube, and CNN+APP2. The compared average high precision and recall values proved that our method can more effectively reduce high noise interferences and achieve more complete and accurate tracings of complex neurons than current methods.

**Table 1:** Quantitative evaluation of the proposed and state-of-art methods on public BigNeuron datasets.

Method	Precision [%]					Recall [%]				
Data	Ours	APP2	neuTube	ST-LVF	CNN+APP2	Ours	APP2	neuTube	STL-VF	CNN+APP2
1	98.9	99.9	98.9	87.2	99.4	94.2	74.0	91.5	92.5	72.3
2	99.2	50.0	99.9	92.0	98.8	97.9	90.4	94.3	94.0	85.6
3	98.3	99.6	99.3	92.6	100	90.7	61.0	82.0	84.4	53.1
4	93.9	98.7	92.6	49.9	100	92.5	44.6	76.6	95.0	29.8
5	98.1	98.8	98.7	44.0	99.6	96.3	79.4	89.7	90.1	69.2
6	99.7	100	97.3	87.7	100	96.0	83.6	95.5	94.8	84.7
7	99.4	79.0	52.2	92.7	95.9	98.0	94.7	99.7	99.1	93.0
8	98.3	100	99.2	87.7	100	95.5	80.3	91.2	87.7	72.9
Average	**98.2**	90.8	92.3	79.2	99.2	**95.1**	76.0	90.1	92.2	70.1
Std	**1.8**	18.0	16.4	20.1	1.4	**2.6**	16.3	7.5	4.6	20.4

*Bold values represent best achieved evaluations and gold standard*.

We also compared the performances of our method and the above methods (Xiao and Peng, 2013; Feng et al., 2015; Li et al., 2017a, 2019b) on fMOST datasets. As shown in Figure 8, the datasets contained a huge amount of unevenly distributed signals with broken parts and high noises. APP2 showed poor performance on these datasets, either over-reconstructed large background noises or hardly traced the broken neurites. neuTube and ST-LVF algorithms wrongly reconstructed some background noises, and led to a number of neurite fragments. The algorithm of CNN+APP2 can prevent the noise interference, while the performances for broken neurites were not good. The proposed method accurately traced these neurites and avoided broken neurites. As shown in Table 2, the average precision and recall were 99.1% and 99.6% respectively for our method, which achieved approximately the same performance as the manual tracing and outperformed current novel algorithms (Xiao and Peng, 2013; Feng et al., 2015; Li et al., 2017a, 2019b) on broken neuron tracing from noisy background.

**Table 2:** Quantitative evaluation of the proposed and state-of-art methods on fMOST datasets.

Method	Precision [%]					Recall [%]				
Data	Ours	APP2	neuTube	ST-LVF	CNN+APP2	Ours	APP2	neuTube	ST-LVF	CNN+APP2
1	99.6	100	96.8	99.5	100	99.6	51.1	93.7	99.4	99.6
2	100	0	73.0	93.6	100	99.4	0	87.2	100	96.3
3	98.9	2.8	83.2	84.0	99.1	100	89.1	87.8	91.6	55.4
4	100	5.5	13.4	17.2	100	100	86.1	61.4	65.9	42.9
5	98.9	93.3	81.7	94.5	100	100	62.8	97.5	85.5	52.9
6	100	0.4	75.9	100	100	100	68.7	86.6	89.2	100
7	93.9	0.4	94.8	100	95.6	97.4	22.3	86.4	62.8	96.4
8	100	100	99.7	99.8	100	100	49.5	91.1	97.2	98.6
9	99.7	5.2	42.0	100	100	99.8	100	97.7	100	100
10	99.8	67.7	78.3	88.4	99.7	100	100	99.7	99.6	99.9
Average	**99.1**	37.5	73.9	87.7	99.4	**99.6**	63.0	88.9	89.1	84.2
Std	**1.9**	46.3	26.8	25.4	1.4	**0.8**	33.2	10.9	14.0	23.6

*Bold values represent best achieved evaluations and gold standard*.

**Figure 9:**
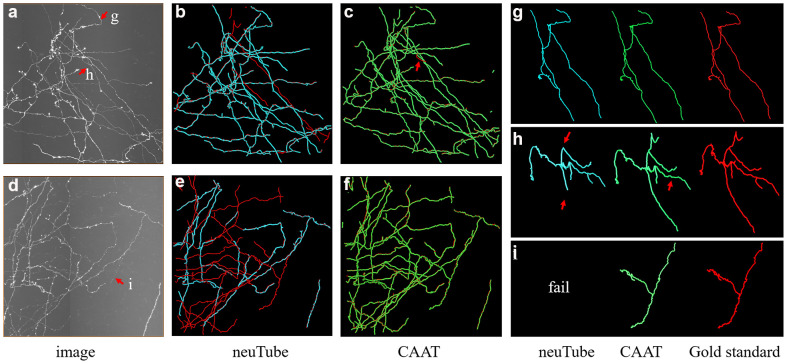
Comparative performances of the proposed method and neuTube algorithms on dense and challenging neuron datasets that contained multiple neurites and various branch structures. Panels **(B,C,E,F)** show the fusion results of automatic algorithms (blue, green) and gold standard (red). Panels **(G–I)** are examples of the traced results that are marked in images **(A,D)**. Inaccurate traced areas were pointed our by arrows.

We exhibited the performance of the proposed method and neuTube method, an automatic tracing algorithm that supports multi-neurite tracing, on some dense and challenging cases that contained dozens of neurites and various branch structures (Figure 9). The experiment was performed on six fMOST image stacks of size 1000 × 1000 × 300. Figure 9 showed two tracing examples, and some tracing results of separate neurites were magnified (Figures 9G–I). neuTube achieved good results on the image with high neurite signals and only missed few inhomogeneous neurites (Figure 9B), while it under-traced lots of dim and discontinuous neurites (Figure 9C). The proposed method nearly traced all these neurites in these neuronal images with both high or dim or uneven neurite signals, and only missed one branch neurite (as pointed out in Figures 9C,H). It could capture most of these complex branch structures. Table 3 shows that the average precision was 96.4% and recall was 99.0% of the proposed method, much better than that of neuTube (precision: 94.1% and recall 79.6%). We also compared the morphological parameter of the two methods. The average path length of the proposed method was 2.58 mm, which was quite similar to that of the gold standard (2.65 mm) and longer than that of neuTube (2.07 mm). The branch point numbers of the proposed method were also similar to the manual tracing result. The neuTube algorithm would trace some neuronal irregularities, and generated too many short and useless branches. For some neurites that were fully overlapped on each other, the proposed method could not separate them and treated them as one neurite. The method is mainly designed for discontinuous neurite tracing, and its continuous tracing results of the dense neuronal image could be used for the subsequent interweaving neurite separation using some novel methods such as NeuroGPS-Tree (Quan et al., 2016) and G-Cut (Li et al., 2019a).

**Table 3:** Quantitative evaluation of the proposed and neuTube methods on some dense neuronal images.

Method	Precision [%]		Recall [%]			Path length [mm]			Branch point number	
Data	Ours	neuTube	Ours	neuTube	Gold standard	Ours	neuTube	Gold standard	Ours	neuTube
1	99.8	98.8	99.3	75.1	2.53	2.46	1.89	19	22	53
2	98.8	95.1	99.3	95.3	2.11	2.03	2.12	25	29	151
3	98.5	95.3	98.3	84.0	2.11	2.02	1.84	22	22	120
4	89.0	85.3	98.5	37.0	3.87	3.80	1.66	45	41	74
5	99.4	98.5	99.2	86.6	3.83	3.75	3.42	51	51	121
6	92.8	91.4	99.4	99.5	1.42	1.43	1.48	8	11	19
Average	**96.4**	94.1	**99.0**	79.6	**2.65**	2.58	2.07	28	29	90
Std	**4.4**	5.1	**0.5**	22.6	**1.00**	**0.98**	0.70	16	14	49

*Bold values represent best achieved evaluations and gold standard*.

**Figure 10:**
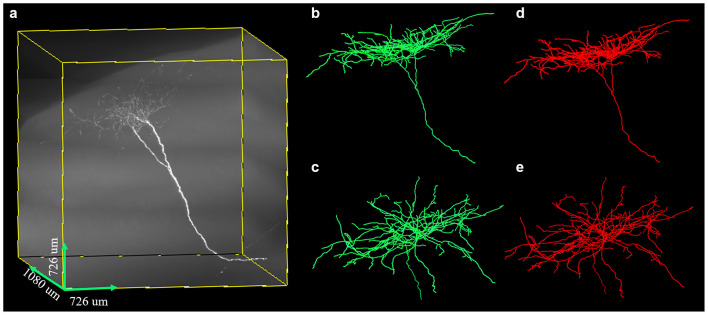
Performances of the proposed method on large-scale neuron data **(A)** with uneven signal and highly branched axons. Panels **(B–E)** are the traced result (green, **B,C**) by the proposed method and gold standard (red, **D,E**) in different 3D views.

We further tested the proposed method on a large-scale fMOST neuronal image. The volume of the dataset was approximate 48 GB and the image size was 3,630 × 3,630 × 1,080. To trace the large data, we first divided the huge volume into image blocks of size 512 × 512 × 512 with overlapped gap of 15 pixels along X, Y, Z direction in sequence, then traced these image blocks, and assembled every two tracing results if they had overlapped skeletons. Figure 10 shows the tracing performance of our method on this challenging image that contained uneven signals along neurite or at neurite intersections and highly branched axons. The proposed method could capture nearly all the neurites and branches of the large data and achieved result similar to manual tracing. The precision and recall of our method on the image were 99.4% and 99.8%. The traced neurite length by manual tracing was 19.4 mm, and by our method was 20.0 mm. As we preserved some short branches of the neuron which were ignored by manual tracing, the path length by our method was a litter longer than that of manual tracing. The accurate performance indicated that our method could be applicable to large-scale neuronal image tracing automatically.

## Discussion

This article presented a fully automatic optical neuronal image tracing method. The proposed method estimated the neurite probability using 3D residual CNN as preprocessing and presented a CAAT algorithm for continuous tracing of complex structures and broken neurites. The main contributions of the article are summarized as follows: (1) an automatic neuron tracing method is presented for precise and complete neuron tracing. (2) A CAAT method is designed to address the difficulty of tracing these complicated and twisted structures and broken neurites that had uneven predicted probability. (3) The proposed method is superior to current novel tracing methods on both public BigNeuron and private fMOST datasets with various structures, distorted and discontinuous neurites.

The tracing of fuzzy and broken neurites with diverse structures from noisy background is common in an optical neuronal image and becomes one of the leading challenges of neuron tracing. Despite numerous previous methods, over-tracing of noises, incomplete tracing of broken neurites and inaccurate tracing of complex neurons are commonplace in neuronal images. We presented an effective neuron tracing method to address these issues, which is achieved by the following aspects: (1) the neurite probability map is robustly and accurately estimated by 3D residual CNN with hybrid loss function and a series of training skills, including various data augmentation. The prediction outperforms current traditional methods in preventing high noise disturbance and estimating neurites (see Figures 3, 7, 8), using more abundant and discriminative features without limitation of a specially designed model or feature. (2) A CAAT method is proposed for continuous tracing of broken neurites with complex structure. The method is based on VS tracing (Rodriguez et al., 2009), which uses the physical spatial location and connectivity property, and can follow the natural structure of object skeleton among various topological structure changes without direction or shape restriction. Thus, the proposed CAAT method showed better performances in neuron tracing at branches and distorted neurites than some novel tracing methods (see Figures 7, 8). The method makes full use of the strong and low probability information for uneven intensity distributed neurite tracing without re-estimating. The prediction of the thin and broken object is an admitted difficult problem in segmentation, the estimation of these objects by 3D CNN was discontinuous as annotators do in uncertain cases. Experiments including repeatedly adjusting network parameters, choosing one best-trained network, and some network modifications might improve the prediction slightly. While these difficult neurites were still hard to be predicted, and a large amount of time and effort was needed for the attempts considering the randomness in network training. The proposed method uses the strong probability information for the accurate position of the main structure of objects, and discontinuous probability information for the continuous tracing in the hard cases. It builds an adaptive tracing term, by combing the characters of distance, region connectivity, and probability continuity along the direction, to determine the connection of discontinuous probability of the difficult tracing. Figures 5, 6 both validated that the CNN prediction and CAAT of the proposed method achieved similar or better performances than current novel enhancement or tracing methods for neuron tracing with quite uneven intensity distribution and close neurites. Figures 7, 8 proved that the performances of the proposed method were more complete and precise than the CNN based VS and CNN+APP2 methods, and better than current tracing algorithms on neuronal image with broken fragments and very complicated structures. Figures 9, 10 further proved that the proposed method could achieve continuous neurite tracing on some challenging cases with dense and large neuronal images. These results indicated the potential of the proposed method for continuous tracing of large–scale neurons with varieties of direction trends and discontinuous neurites.

Though the proposed method showed high performance on neurite tracing, it has some limitations. The tracing would not separate some crossed objects due to the dense distribution and low resolution. In the future, we will test the proposed method on more neuronal datasets for robustness evaluation. We will further improve the prediction result for individual object segmentation and study the logical criteria for overlapped objects to promote the tracing in a dense area.

## Conclusion

We propose a fully automatic and precise neuron tracing method for optical images. A 3D deep residual CNN with a series of training skills was applied for accurate neurite probability estimation as preprocessing. A CAAT method was proposed for continuous tracing of complicated and discontinuous neurites, using the distance, connectivity, probability, intensity, and continuity properties of the broken fragments. The performances on the public BigNeuron datasets and private fMOST datasets proved our method can accurately trace these broken neurites with complex structures. Our method achieved similar results as manual performance and was superior to current novel tracing methods. The precise tracing of optical images proved the potential of our method for continuous neuron tracing on a large-scale.

## Data Availability Statement

The datasets presented in this study can be found in online repositories. The names of the repository/repositories and accession number(s) can be found below: https://github.com/GTreeSoftware/Neuron-Tracing-CAAT/releases/tag/1.01.

## Author Contributions

SZ and TQ conceived the project. QH designed the algorithm and wrote the manuscript. TQ and SZ corrected the manuscript. YC and TC performed image analysis. SZ and AL produced the dataset. All authors contributed to the article and approved the submitted version.

## Conflict of Interest

The authors declare that the research was conducted in the absence of any commercial or financial relationships that could be construed as a potential conflict of interest.

## Publisher’s Note

All claims expressed in this article are solely those of the authors and do not necessarily represent those of their affiliated organizations, or those of the publisher, the editors and the reviewers. Any product that may be evaluated in this article, or claim that may be made by its manufacturer, is not guaranteed or endorsed by the publisher.
